# *NLRP3* Inflammasome as a Therapeutic Target for Atherosclerosis: A Focus on Potassium Outflow

**DOI:** 10.31083/j.rcm2308268

**Published:** 2022-07-22

**Authors:** Yi-Jing Jin, Zhuo-Yu An, Zhi-Xuan Sun, Xin-Chen Liu

**Affiliations:** ^1^Peking University Health Science Center, 100191 Beijing, China; ^2^Department of Cardiology, Peking University First Hospital, 100034 Beijing, China; ^3^Peking University Institute of Hematology, Peking University People's Hospital, 100044 Beijing, China; ^4^Peking University Third Hospital, 100191 Beijing, China

**Keywords:** atherosclerosis, *NLRP3* inflammasome, potassium efflux, therapeutic target

## Abstract

Atherosclerosis is a risk factor for various cardiovascular diseases, and is linked to high rates of morbidity and mortality across the globe. Although numerous complex processes are involved in the development and progression of atherosclerosis, the exact mechanisms behind its pathogenesis remain unclear. Inflammation and endothelial cell damage exert a lasting effect on atherosclerosis, causing lipid and fibrous tissue accumulation in the intima of the artery to form plaques, and subsequently promoting atherosclerosis. Nod-like receptor protein 3 (*NLRP3*) inflammatory corpuscle is thought to be the link between lipid metabolism and inflammation. Long Potassium outflow is a vital activator of *NLRP3*, with a simultaneous effect as a start-up and adjustment. The majority of existing drugs for atherosclerosis targeting the *NLRP3* signaling pathway target IL-1, whereas drugs targeting the critical link of potassium efflux are relatively new. This review discusses the *NLRP3* inflammatory corpuscle as a critical regulator of the immunological inflammatory pathway in atherosclerosis. Moreover, current knowledge on *NLRP3* inflammatory corpuscle start and activation pathways were integrated, emphasizing potassium-involved outflow-related proteins. We highlight potential treatment approaches for *NLRP3* inflammatory corpuscle pathways, specifically targeting potassium outflow channels of targeted drugs. Collectively, these insights indicate that targeting the *NLRP3* inflammatory corpuscle is a vital anti-inflammatory therapy for treating atherosclerosis.

## 1. Atherosclerosis

Atherosclerosis is the most prevalent and important pathological condition in atherosclerotic vascular diseases, causing various cardiovascular diseases (CVDs), including coronary heart disease, hypertension, stroke, etc. These diseases constitute the global leading cause of death. In 2019, studies estimated that 17.9 million people succumbed to CVD, accounting for 32% of all global mortalities. Notably, 85% of these were attributed to coronary heart disease and stroke [1]. At present, CVD etiology remains unknown, due to a combination of multiple risk factors, including dyslipidemia, hypertension, diabetes, smoking, genetics, and obesity [2].

Atherosclerosis is thought to be caused by endothelial dysfunction and dysregulation of circulating lipid metabolism [3, 4, 5]. During its pathology, the earliest identifiable changes include focal deposition and oxidative modification of circulating lipoprotein particles dominated by low-density lipoprotein (LDL) under the endothelium [6]. After endothelial damage, circulating monocytes are selectively recruited into the endangium, where they differentiate into macrophages to eliminate the deposited lipoproteins. The deposited lipoproteins engulf the modified lipoproteins and become foam cells, forming early lipid streaks. An increase and decrease in circulating LDL and HDL levels, respectively, cause the formation of numerous foam cells. Consequently, smooth muscle cells in the tunica media vasorum are recruited to migrate. Migrating smooth muscle cells engulf the lesions that cannot be cleared. Under the stimulation of various cytokines, they generate collagen fibers, elastic fibers, and other fibrous caps wrapping the necrotic foam cells, forming typical atherosclerotic plaques. Notably, inflammation plays an indispensable role in the abovementioned atherosclerosis process [7, 8].

Recognized risk factors, including smoking, hypertension, and obesity, among others, exacerbate the production of reactive oxygen species (ROS). Previous studies indicate that angiotensin II activates monocytes, releasing pro-inflammatory cytokines [9, 10]; nicotine causes inflammation in the endothelium [11, 12], whereas metabolic disorders, including increased visceral fat and insulin resistance, promote the release of inflammatory mediators [13]. At the same time, the abovementioned risk factors are involved in increased LDL levels [14, 15, 16, 17]. Through ROS accumulation in the intima of blood vessels, LDL undergoes oxidative modification to generate OX-LDL, which subsequently causes inflammation of the blood vessel wall by binding toll-like receptor (TLR) and scavenger receptor. Therefore, OX-LDL is a clinical marker of plaque inflammation. OX-LDL produces chemotactic intercellular adhesion molecule-1 (ICAM-1) and vascular cell adhesion molecule-1 (VCAM-1), which enhance the adhesion properties of endothelial cells and promote the binding of monocytes to endothelial cells. Consequently, inflammatory cells and monocytes release monocyte chemoattractant protein-1 (MCP-1) to activate leukocytes and stimulate smooth muscle cell proliferation [18]. At the same time, cholesterol load also forms intracellular cholesterol crystals, which subsequently activate inflammasomes, and enhance the expression and release of numerous proinflammatory cytokines [19]. Previous experimental findings have shown that the deletion of inflammatory genes minimizes the risk of atherosclerosis development. During the late stages of atherosclerosis development, inflammatory cell secretions of matrix metalloproteinases (MMPs) become degraded patches of collagen fibrous caps rich in bursting lipid plaques. However, the necrotic tissue factor of the core is exposed to circulation in the blood, activating the blood coagulation cascade reaction, and causing blood clot formation as well as the development of tissue ischemia [20].

Considering the aforementioned pathogenesis, lowering LDL levels is currently considered a basic treatment approach for atherosclerosis in clinical practice. Although effective methods including statins and preprotein invertase *Bacillus subtilis* invertase/Kexin9 (PCSK9) inhibitors inhibit the levels of circulating LDL to a certain extent, they have been linked to various adverse cardiovascular events, threatening the lives of patients [21]. For instance, a 2017 CANTOS trial [22] revealed that although canakinumab, a monoclonal antibody that inhibits *IL-1β* reduced the incidence of adverse cardiovascular events (MACEs), it significantly exacerbated the incidence of coinfections and sepsis. Moreover, studies have confirmed that anti-inflammatory methods, including colcoline, methotrexate, methotrexate (a monoclonal antibody that blocks the *IL-6* receptor) [23], and ApoB peptide inoculation that causes Tregs [24], have either therapeutic or preventive effects on atherosclerosis. Thus, inhibiting inflammatory response is a novel and potential therapeutic strategy for preventing atherosclerotic thrombotic events, improving and complementing the current lipid-lowering therapies without bleeding complications.

## 2. *NLRP3* Inflammasome

The classic inflammasome refers to a polymeric protein complex, primarily comprising sensor proteins, junction molecules, and effectors. Typical inflammasome sensor proteins include nucleotide-binding oligomerization domain (NOD), leucine-rich repeat (LRR) sequence receptors *NLRP1*, *NLRP3*, *NLRP6*, *NAIP*/*NLRC4*, melanoma-2 (AIM2)-like receptors and *PYRIN*, a protein containing triangular motif (*TRIM*) [25]. Each of the above responds to specific pathogen-associated molecular patterns (PAMPs) or danger-associated molecular patterns (DAMPs). The connector molecule is the apoptosis-associated speck-like protein *ASC*, with a caspase recruitment domain (CARD). This complex recruits effector cysteine proteases that generate inflammatory factors, including *IL-1β*, *IL-18*, and *IL-37 * [26], and activate pore-forming gasdermin D (GSDMD) to induce apoptosis.

*NLRP3* comprising an N-terminal pyrin domain (PYD), a central ATPase domain (*NACHT*), and a C-terminal LRR primarily exists in inflammatory cells activated by inflammatory stimulation, including macrophages, monocytes, dendritic cells, and splenic neutrophils. Also, it is expressed in bone marrow, muscle, endocrine cells, and neurons. *NLRP3* can either be activated by PAMPs or DAMPs to open the PYD and interact with the PYD in *ASCs*. Moreover, the CARD on ASCs combines with that on procaspase-1. Collectively, these substances integrate, making up the *NLRP3* inflammasome [27]. *NLRP3* inflammasome formation causes self-cleavage of procaspase-1, generating an active caspase-1p10/p20 tetramer, which subsequently cleaves the cytokine precursors pro-*IL-1β* and pro-*IL-18*, causing them to mature, be released, and an inflammatory-associated cell death known as pyroptosis [28]. Therefore, the inflammasome is a critical signaling platform during defense against pathogens. The inflammasome helps to eliminate damaged host cells and stimulates the adaptive immune response. Previous studies have implicated abnormal activation of the inflammasome in the development of several inflammatory diseases, such as type 2 diabetes, atherosclerosis, Alzheimer’s disease (AD), infectious diseases, cancer, and autoimmune diseases. Additionally, inflammasomes synthesize eicosanoids, thereby antagonizing each other with autophagy, promoting phagosome maturation in cells, and regulating metabolism [29].

### 2.1 Activation and Regulation of the NLRP3 Inflammasome

**Fig. 1.**
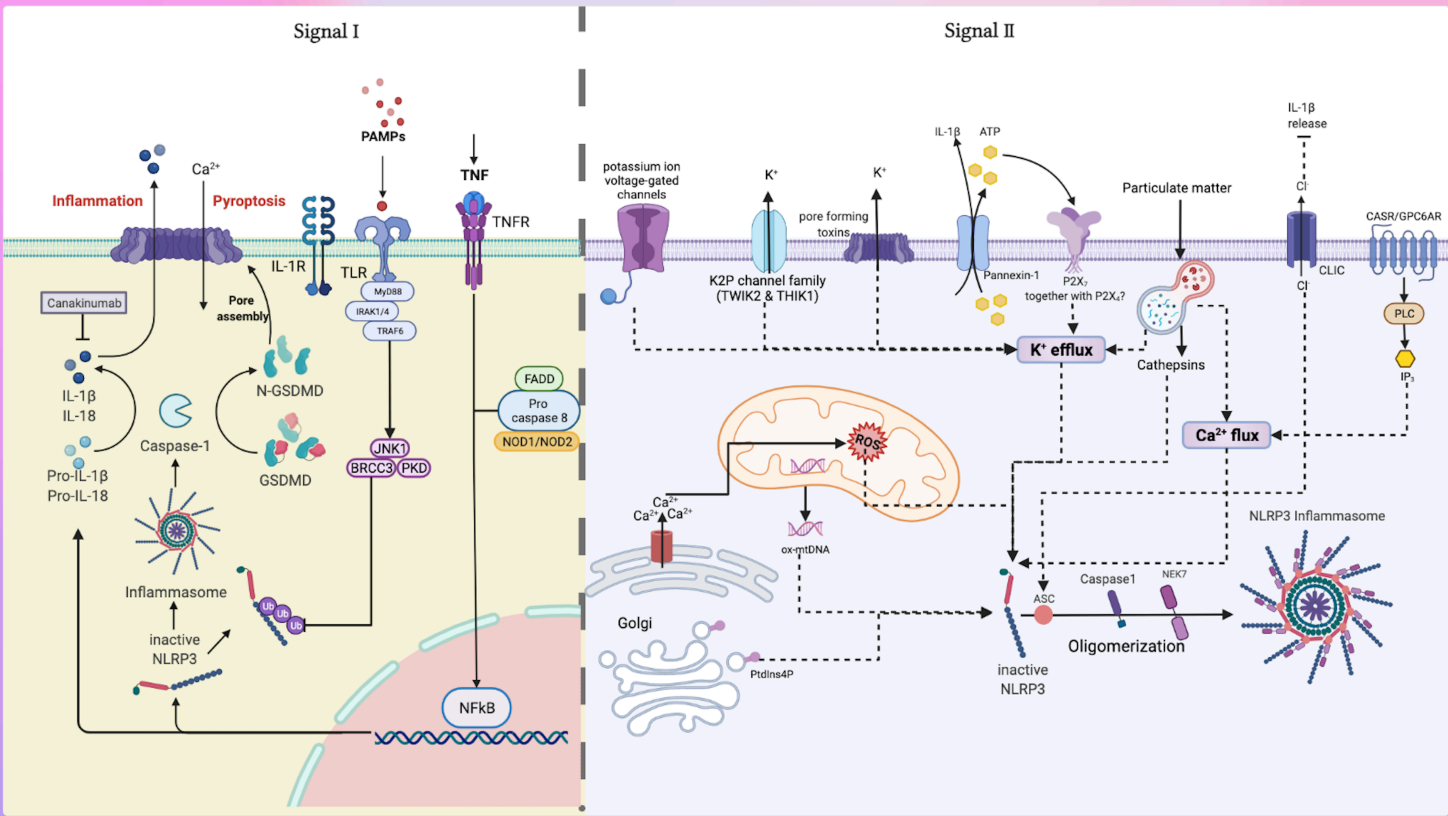
**Classic activation pathways and common drug targets of NLRP3 inflammasome**. The classic activation pathway of NLRP3 inflammasome is a two-signal model. Signal I is induced by PAMP or DAMP stimulation of TLR and NLR, which activates NF-κB and up-regulates the expression of NLRP3 and pro-IL-1β. BRCC3 also mediates K63 related NLRP3 deubiquitination and promotes the activation of NLRP3 inflammasome. Signal II activates the assembly of NLRP3 inflammasome by a variety of events, including ion flux, mitochondrial oxidative damage, lysosomal membrane rupture, endoplasmic reticulum activation, metabolic disturbance, and trans-Golgi decomposition, which activates ASC, Pro-caspase-1 and NLRP3 oligomerization to form NLRP3 inflammasome. The formation of NLRP3 inflammasome triggers the self-cleavage of procapase-1 to produce active caspase-1, which cleaves the cytokine precursors pro-IL-1β and pro-IL-18, causing them to mature and secrete, and it also cuts off the N-terminal domain of GSDMD to create pore in the cell membrane, triggering inflammation-related cell death, known as pyroptosis. PAMP, pathogen-associated molecular patterns; DAMP, damage-associated molecular pattern; TLR, toll-like receptor; NLR, NOD-like receptor; TNF, tumor necrosis factor; FADD, fas-associated protein with death domain; MyD88, myeloid differentiation primary response protein 88; IRAK, IL-1 receptor associated kinase; TRAF, TNF receptor associated factor; JNK1, c-Jun N-terminal kinase-1; PKD, protein kinase domain; BRCC3, BRCA1/BRCA2-containing complex subunit-3; GSDMD, gasdermin D; CLIC, intracellular chloride channel; CASR/GPC6AR, calcium-sensing receptor; PLC, phospholipase C; IP3, inositol triphosphate; Nek7, Serine/threonine-protein kinase-7; ASC, apoptosis-associated speck-like protein containing a CARD; ROS, reactive oxygen species; ox-mtDNA, oxidized mitochondrial DNA; Ptdlns4P, phosphatidylinositol 4-phosphate.

At present, *NLRP3* inflammasome activation is considered a two-signal model comprising prime activation and activation. In this model, *NLRP3*, pro-*IL-1β,* and pro-*IL-18* expression are upregulated by either microbes or endogenous cytokines, providing the first signal (Fig. 1). The second signal is generated by extracellular ATP, pore-forming toxins, and particulate matter, which promote inflammasome assembly and the lysis of pro-caspase-1 to form active caspase-1, which in turn lyses pro-*IL-1β* and pro-*IL-18* to release mature *IL-1β* and *IL-18*. Previous studies indicate that this process is also regulated by multiple *NLRP3* posttranslational modifications and interacting substances [30].

The inflammasome constitutes the innate immune system. Notably, Signa I is activated by pattern recognition receptors (PRRs) recognizing harmful stimuli, including PAMPs and DAMPs. PAMPs include common bacterial cell wall components, viral products, and bacterial cell nucleus components, which primarily include sugars and lipids, whereas DAMPs comprise endogenous cytokines released after stimulation of tissues or cells by damage, hypoxia, stress, and other factors. Atherosclerosis is mostly mediated by DAMPs. Harmful stimulation of TLR, NLR, and cytokine receptor macrophages activates the transcription factor *NF-κB*, hence upregulating the expression of inactive *NLRP3* and mediating the non-constitutive expression of pro-*IL-1β*. Nonetheless, the concentration is inadequate to activate inflammasome assembly under resting conditions. Furthermore, this phenomenon does not influence ASC expression, pro-caspase-1, and pro-*IL-18*. Additionally, MyD88, TRIF, and other signaling molecules in the *NF-κB* signaling pathway regulate the expression of *NLRP3* and pro-*IL-1β*. Previous studies have shown that FADD and caspase-8 also trigger *NLRP3* expression during prime activation [31]. Notably, posttranslational modifications of *NLRP3*, including phosphorylation and ubiquitination modulate prime activation of the *NLRP3* inflammasome. Furthermore, prime activation requires activation of both IRAK1 and proteasome-dependent ERK, processes involving posttranslational modifications of *NLRP3* as follows: (1) In resting macrophages, *NLRP3* is ubiquitinated by a mixture of K48 and K63 ubiquitin chains, thereby maintaining its inhibited condition; (2) ABRO1 recruits BRCC3 to mediate K63-related *NLRP3* de-ubiquitination, promoting prime activation of *NLRP3* inflammasome [32]; (3) LUBAC mediates ASC ubiquitination; and (4) SYK and JNK mediate the ASC phosphorylation [33]. Signal II activates *NLRP3* inflammasome assembly, a process involving numerous events, including ion flux, mitochondrial oxidative damage, lysosomal membrane rupture, endoplasmic reticulum activation, metabolic disturbances, and trans-Golgi breakdown.

### 2.2 Non-Canonical NLRP3 Activation

Besides the aforementioned classical two-signal transcriptional activation pathways, the critical role played by noncanonical and alternative activation of the *NLRP3* inflammasome has been documented (**Supplementary Table 1**). Specifically, the non-canonical *NLRP3* inflammasome activation pathway is mediated by caspase-11 in mouse cells or caspase-4/caspase-5 in human cells in response to LPS in gram-negative bacteria. In humans, caspase-4 is constitutively expressed in numerous non-monocytes and monocytes. Therefore, cytoplasmic LPS activates non-classical inflammasomes without priming steps [34]. In this pathway, extracellular LPS activates *TLR4*, induces type Ⅰ interferon response, and complements the C3-C3aR axis, thereby upregulating caspase-11 expression. Previous findings indicate that Caspase-11 directly recognizes lipid A in the conserved structure of cytoplasmic LPS, causing its oligomerization and automatic proteolysis [35]. This process is followed by gasdermin D (GSDMD) generation, which causes cell lysis and pyrosis [36], and ATP release from pannexin-1 [37]. Consequently, this activates the P2X7 receptor, resulting in potassium outflow, activating *NLRP3*-caspase-1-dependent *IL-1β* secretion. Overall, this suggests an interaction between classical and non-classical inflammasome activation pathways.

An alternative inflammasome activation pathway occurs in human monocytes and secretes *IL-1β* independent of the classical inflammasome activation pathway. This phenomenon has not been observed in mice. Also, this pathway is signaled by LPS-stimulated TLR4 without second stimulation, indicating its potassium independence. Although NLRP3, ASC, and caspase-1 can be generated via the TRIF-RIPK1-FADD-caspase-8 pathway, no pyroptosomes are formed due to the absence of both the second signal, and pyroptosis. Nevertheless, mature *IL-1β* is released [38]. Studies found that caspase-8 mediated pathway can also occur in iNKT cell culture *in vitro*, although its upstream signal is different and is potentially mediated by the TNFR family on the cell membrane [39]. In addition to LPS, *NLRP3* activation mediated by oxPAPC (an endogenous oxide membrane lipid) [40] and hexokinase [41] is also characterized by alternative activation. However, the interaction among the three activation pathways remains unknown. Previous studies have shown that alternative activation pathways do not induce pyroptosis, but potentially promote homeostasis of antigen transport to secondary lymphoid organs and resident bone marrow cells [42]. A recent study revealed that even in the absence of the priming step, human mononuclear cells assemble functional *NLRP3* inflammasomes *in vitro* in response to the activation signal, causing inflammation during the early stage of atherosclerosis [43]. However, the underlying mechanism remains unclear so far, although monocyte-based startup states could allow inflammasomes to quickly assemble and cope with all types of danger signals.

### 2.3 Role of Potassium in NLRP3 Inflammasome Function

*NLRP3* inflammasome activation involves various ion flux events, including potassium outflow, calcium mobilization, chloride outflow, and sodium inflow. Among them, is potassium outflow, which functions upstream of *NLRP3* inflammasome activation; this is extensively considered a necessary condition and common feature of classic *NLRP3* inflammasome activation. That is, potassium outflow occurs before *NLRP3* inflammasome activation. Studies have shown that high extracellular potassium also inhibits *NLRP3* inflammasome activation [44], whereas other scholars indicate that *NLRP3* inflammasome activation is unrelated to potassium outflow [38, 45]. Despite extensive research, the specific molecular mechanisms by which potassium outflow causes *NLRP3* inflammasome activation to remain poorly understood. Elsewhere, researchers have hypothesized that potassium outflow may be associated with conformational changes in *NLRP3*, mitochondrial dysfunction, and mtROS production that promote *NLRP3* inflammasome activation [46].

Previous studies indicate that the P2X7 receptor, pannexin-1, K2P channel, and GSDMD are closely related to this process [47]. Additionally, LPS binds to complement components *in vivo*, forming membrane-attacking complexes on the cell membrane. On the other hand, C3a binds to receptors on monocytes to release ATP, whereas particle irritants, including alum, silica crystals, and calcium pyrophosphate crystals directly cause the outflow of potassium ions [48]. The P2X7 receptor is an ATP-gated non-selective cation channel with a pore-forming motif similar to that of a potassium ion channel, that can be activated by extracellular ATP for direct outflow of potassium ions. Its deficiency is linked to the inhibition of *IL-1β* maturation [49], a phenomenon that has made researchers speculate that the potassium outflow channel mediates *NLRP3* inflammasome activation. Long-term activation of the P2X7 receptor ion channel causes a continuous expansion of its ion pore, accompanied by a continuous increase in cell membrane permeability. Notably, it allows the passage of molecules the size of up to 900 kDa, causing an outflow of intracellular potassium ions [50]. Previous research evidence also demonstrated that P2X4 receptors promote *NLRP3* activation with P2X7. Besides, P2X4 receptors alone constitute a novel pathway of *NLRP3* activation, which may be caused by P2X4 receptor deficiency, resulting in reduced P2X7 receptor expression [51].

Of note, pannexin-1 is a non-selective macroporous channel, whose relationship with the *NLRP3* inflammasome remains puzzling. Nonetheless, it is closely associated with apoptosis. Previous studies indicate that annexin-1 releases *IL-1β* in response to ATP and nigericin [52]. At the same time, pannexin-1 may mediate cellular ATP release, hence promoting P2X7 receptor signaling [53]. Also, studies have shown that accumulated triglycerides increase extracellular ATP through pannexin-1, hence activating ATP-sensitive potassium channels and caspase cascade reaction, promoting potassium outflow, as well as causing macrophage apoptosis, and plaque instability [54]. Moreover, experimental results have shown that caspase-1-mediated pyroptosis form GSDMD, thereby causing potassium outflow.

The two-pore domain potassium (K2P) channels are an important family of mammalian potassium channels that maintains the resting membrane potential in nearly all cells. Previous studies indicate that TWIK2, a member of the K2P channel family, has a synergistic effect with P2X7 in macrophages. The former causes calcium and sodium ions to flow in to change the membrane potential, whereas the latter causes potassium ions to flow out to activate the *NLRP3* inflammasome. THIK1 is another member of the K2P channel family that activates the *NLRP3* inflammasome in microglia [55]. Also, potassium ion voltage-gated channels KCNA3 and KCNB2 as well as potassium ion inward rectifier channels KCNJ3, KCNMA1, and KCNN4 are involved [56].

Nek7 is a member of the mammalian NIMA-related kinase (*Nek*) family recognized as an essential mediator in downstream activation of the *NLRP3* inflammasome by potassium outflow. It is a multifunctional kinase that influences processes, including centrosome replication, mitochondrial regulation, intracellular protein transport, DNA repair, and mitotic spindle assembly. For instance, He *et al*. [57] reported that as an NLRP3 binding protein, *Nek7* acts downstream of potassium ion outflow and regulates *NLRP3* oligomerization and activation. In the absence of *Nek7*, caspase-1 activation and *IL-1β* release are suppressed only in *NLRP3* in response to activation signals. The authors also found that *Nek7* majorly interacts with NOD and LRR of NLRP3, improving the positive feedback. Also, previous studies have shown that potassium ion outflow promotes this process. In 2019, one study [58] further revealed that *NLRP3* should first be bound to *Nek7*. Secondly, the *NLRP3*-*NEK7*complex formation may be inadequate to activate *NLRP3* since the oligomerization requires *NACHT* conversion from an inactive to an active conformation; this may necessitate ATP binding and other unknown steric triggers.

### 2.4 Relationship between the NLRP3 Inflammasome and Atherosclerosis

Previous research findings have shown that atherosclerosis is globally recognized as a chronic inflammatory disease. Moreover, its course is nearly free of microbial infection; thus, atherosclerosis-associated inflammation is frequently considered an aseptic inflammation [59, 60]. Aseptic inflammation is majorly caused by inflammasome activation, among which studies on the *NLRP3* inflammasome have reached maturity. Clinical and basic study results have shown that the *NLRP3* inflammasome is expressed in endothelial cells, immune cells, smooth muscle cells, and other cells involved in the pathogenesis of atherosclerosis. However, its products *IL-1β* and *IL-18* also influence the occurrence and size of atherosclerotic plaques [61, 62]. Results from several epidemiological studies indicate that *NLRP3* inflammasome activation is linked to the development of atherosclerosis in humans [61, 63, 64, 65]. For instance, *NLRP3* was significantly upregulated in the aorta of CABG patients and non-atherosclerotic participants, with its expression levels significantly associated with coronary artery severity [66]. In patients with the acute coronary syndrome, *NLRP3*, *IL-18* precursor, *IL-18* and *IL-1β* levels were higher in subjects with acute myocardial infarction and angina pectoris and correlated with serum total cholesterol, LDL, and OX-LDL levels, relative to normal subjects. Moreover, *NLRP3* positively correlated with downstream inflammatory mediators [67].

**Fig. 2.**
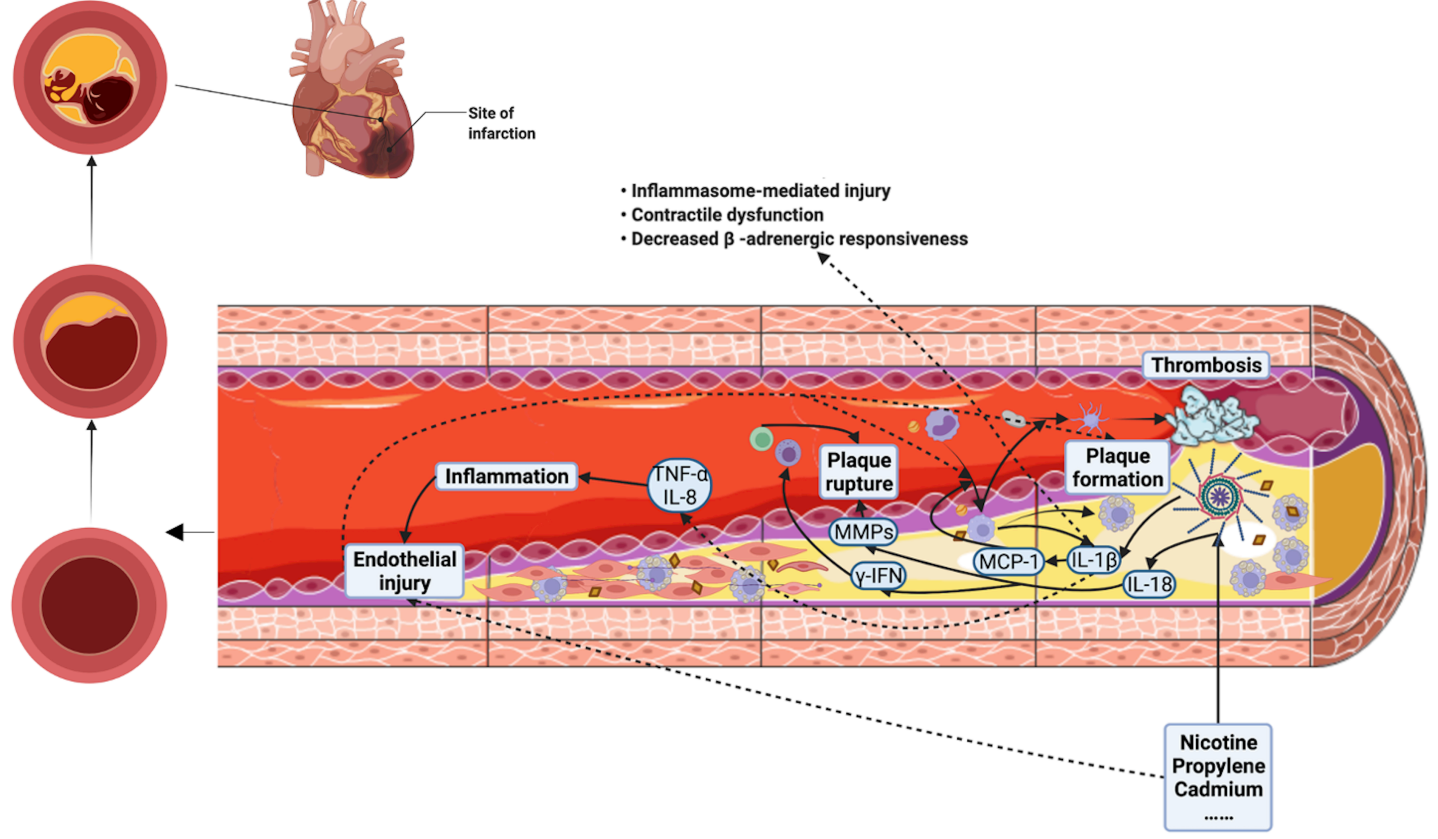
**Relationship between NLRP3 inflammasome and atherosclerosis and acute myocardial infarction**. NLRP3 inflammasome plays a role in promoting different stages of atherosclerosis: (1) During plaque formation, NLRP3 inflammasome can destroy endothelial cells directly by itself or by causing inflammatory reactions, leading to deposition of ox-LDL and cholesterol, while IL-1β released by NLRP3 inflammasome causes MCP-1 production, recruitment of monocytes, and formation of foam cells; (2) During plaque rupture, NLRP3 inflammasome releases IL-18, which in turn produces MMPs and γ-IFN (recruiting NK cells and T cells) to cause plaque rupture; (3) Macrophages also activate platelets to form thrombosis, further aggravating vascular occlusion. NLRP3 inflammasome exacerbates atherosclerotic lumen narrowing, which can cause acute myocardial infarction. In addition, NLRP3 inflammasome can directly promote the different processes of acute myocardial infarction by directly inducing myocardial cell apoptosis and reducing β adrenergic reactivity. ox-LDL, oxidized low density lipoprotein; IL-1β, interleukin-1β; MCP-1, monocyte chemotactic protein-1; IL-18, interleukin-18; MMPs, matrix metalloproteinases; γ-IFN, γ-interferon; IL-8, interleukin-8; TNF-α, tumor necrosis factor-α.

Although the role of *NLRP3* in atherosclerosis has not been fully established, several studies have shown that it modulates the early stages of disease development. Endothelial injury is the first step of atherosclerosis, whereas expression of *NLRP3* and ASC in endothelial cells increases under the action of nicotine, ultimately causing pyroptosis [11]. The vascular ECs cover the intima of blood vessels, forming a semipermeable barrier between circulating blood and the extravascular matrix. *IL-1β*, *IL-18*, and *HMGB1* released by *NLRP3* activation activate the *NF-κB* signaling pathway, which in turn promotes transcriptional activation of chemokines and adhesion molecules, thereby increasing leukocyte adhesion, and disrupting cell permeability. Various pollutants, including ${\mathrm{PM}}_{2.5}$ [68], acrolein [69], and cadmium are involved in *NLRP3* inflammasome activation. This causes an increase in the level of serum inflammatory cytokines and damage to endothelial cells, aggravating the formation of atherosclerotic plaques. After endothelial dysfunction, monocytes adhere to the lesion site, differentiating into macrophages (Fig. 2) [70].

Several pieces of evidence have shown that *NLRP3* inflammasome activation enhances lipid deposition and migration in macrophages as well as accelerates foam cell formation [71]. As mentioned above, macrophages phagocytosing ox-LDL or cholesterol to form foam cells are associated with *NLRP3* in the following ways [72]: (1) lysosomes release ROS and proteases to activate *NLRP3*; (2) TLRs recognize ox-LDL and free fatty acids to induce NF-κB expression, which upregulates *NLRP3* expression and *IL-1β* precursors; (3) *IL-1β* mediates upregulation of monocyte chemotactic protein (MCP-1), which recruits monocytes to activate platelets and promote their release; and (4) IL-18 causes necrosis of vascular smooth muscle cells, releasing tissue metalloproteinases, and reducing plaque stability. Duewell *et al*. [61]. Revealed that transplantation of *NLRP3*-deficient bone marrow cells into LDL receptor-deficient mice predisposed to atherosclerosis is associated with inhibition of atherosclerosis. Also, other studies have shown that *NLRP3*, ASC, *IL-1β*, *IL-18,* and other *NLRP3* inflammasome components, with their respective products, are significantly expressed in patients with atherosclerosis [65]. Immunostaining results revealed that *NLRP3* and ASC were colocalized with CD68-positive macrophages. Cholesterol crystals trigger *IL-1β* release in human plaques. Pyroptosis is an inflammatory cell death process, where the N-terminal domain of GSDMD is cleaved by active caspase-1 to form pores on the cell membrane; this process has been linked to lysis and cell death [73]. This promotes the development of atherosclerosis by exacerbating inflammation. Nonetheless, the *NLRP3* inflammasome can also trigger apoptosis by activating caspase-8 in macrophages, although it remains unknown whether this function hinders the development of atherosclerosis. Previous studies have also shown that the *NLRP3* inflammasome promotes atherosclerosis progression by targeting a series of cellular and molecular components, including STAT, MAPK, JNK, microRNA networks, ROS, PKR, etc. [74]. In summary, *NLRP3* causes plaque formation and thrombosis and influences its stability [70].

### 2.5 NLRP3 Inflammasome in Acute versus Chronic Inflammation and in Sterilized versus Non-Sterilized Inflammation

Acute inflammation is often caused by PAMPs (infection, non-sterilized inflammation) and DAMPs (cellular stress, trauma). This process occurs over a short period and is characterized by severe manifestations. Previous studies have identified *IL-6*, *TNF-α*, *IL-1β*, and CRP as markers of acute inflammation [75, 76]. The *NLRP3* inflammasome is crucial in *IL-1β* production, which mediates neutrophil infiltration to inflamed sites [77]. Several studies indicate that *NLRP3* inflammasome activation protects against various infections, including *Candida albicans * [78], influenza virus [79], and sepsis [76]. In SARS-CoV-2 infection, the *NLRP3* inflammasome is linked to excessive inflammatory responses via mitochondrial dysfunction and increased *IL-1β* levels [80]. Acute inflammation often results in tissue repair, with fibrous connective tissue hyperplasia and scar tissue formation. One recent study discovered that age-dependent activation of the *NLRP3* inflammasome is related to *TGF-β* activation and ECM deposition in a mouse model [81].

During the normal inflammatory response, inflammatory response subsides upon removal of the stimulus. Nonetheless, acute inflammation is regulated by social, psychological, environmental, and biological factors, which impede its regression and instead promote the development of chronic inflammation in a low-level, non-infectious (i.e., sterile) condition [75]. Chronic inflammation, usually caused by DAMPs, including metabolic disorders, and tissue damage, among others, is age-related causing persistent damage to an organism. Chronic inflammation characterized by metabolic disorders, including type 2 diabetes (T2D), obesity, atherosclerosis, and AD, promote diabetes occurrence, whereas *NLRP3* inflammasome mediates obesity-induced inflammation and insulin resistance [82]. Previous studies have shown that molecules, including high glucose, islet amyloid polypeptides, saturated fatty acids, and mitochondrial ROS activate the *NLRP3* inflammasome and promote T2D pathogenesis [82, 83, 84]. Additionally, Aβ peptide deposition causes chronic inflammation in AD, whereas Aβ-induced activation of the *NLRP3* inflammasome causes the synthesis of neurotoxic factors in microglia, ultimately resulting in AD development [84].

In conclusion, inflammasomes play a double-edged sword in inflammation, whereas their activation in acute inflammation helps in eliminating necrotic cells and initiating tissue repair. Nevertheless, sustained activation of inflammasomes in chronic disease is detrimental, resulting in metabolic disorders and damage to tissues [85].

## 3. Drug Targets Related to the NLRP3 Inflammasome

Through extensive pathogenicity analysis, scholars have confirmed that atherosclerosis is a chronic inflammatory disease. The inflammasome plays a crucial role in this process, specifically with the *NLRP3* inflammasome being extensively studied. Consequently, there has been an emergence of multiple targeted therapies targeting *NLRP3* inflammasome complex cascade signals [86]. These include inhibition of *NLRP3* inflammasome activation and upstream signaling, blocking inflammasome assembly, inhibition of caspase-1 activation, blocking GSDMD cleavage, and neutralization of inflammatory cytokines. Although specific inhibitors of the *NLRP3* inflammasome have significant therapeutic potential, no drugs have so far been approved for direct *NLRP3* inflammasome inhibition.

### 3.1 Drug Targets Associated with Potassium Outflow

#### 3.1.1 Glibenclamide

Only a few drugs targeting potassium channels in *NLRP3* inflammasome activation have been developed. The sulfonylurea compound glibenclamide is an extensively studied compound targeting potassium channels. Glibenclamide is an oral hypoglycemic drug with anti-inflammatory effects through *NLRP3* inflammasome inhibition, reduction of proinflammatory cytokine production, and inflammatory cell recruitment as well as inhibition of NO production. This drug has therapeutic effects against respiratory, digestive, urinary, heart, and central nervous system inflammatory diseases as well as ischemia-reperfusion injury processes [87]. Glibenclamide is an ATP-sensitive potassium channel ($K_{\text{ATP}}$) blocker and a broad-spectrum ATP binding box transporter (ABC) inhibitor. $K_{\text{ATP}}$ channel comprises four pore-forming subunits (KIR6.x) and four regulatory sulfonylurea receptor (Sur) subunits highly expressed in atherosclerotic macrophages, particularly Sur2A and Kir6.2, with *TNF-α* overexpression [88]. Studies have reported that $K_{\text{ATP}}$ is not involved in *NLRP3* inflammasome inhibition [89]. This is confirmed by inhibiting inflammasome activation in macrophages lacking the $K_{\text{ATP}}$ subunit by glibenzoide, and Sur1 inhibition by glipizide but not *NLRP3* inflammasome activation. Glibenclamide decreases levels of LPS-induced *TNF-α* and abrogates *INF-γ* release by inhibiting ATP and P2X7 receptors. P2X7 receptor activation is dependent on membrane potential, modulated by the Kir6.2 subunit of the $K_{\text{ATP}}$ channel expressed on monocytes. Studies indicate that morphine trigger neuronal release of HSP70 via the MOR/AKT/KATP/ERK pathway in microglia [90]. This provides an alternative pathway for *TLR4* signaling activation. Moreover, glibenclamide inhibits HSP70 release and *NLRP3* inflammasome activation by blocking the $K_{\text{ATP}}$ channel.

#### 3.1.2 Berberine

Different specific agents for P2X7 receptor and NLRP3 inflammasome inhibition have been developed. None of these drugs is currently approved for therapeutic use; a few are at early clinical stages. Berberine is a bioactive base extracted from various herbal components with enormous pharmacological effects, including antibacterial, anticancer, anti-inflammatory, blood-glucose-lowering, and lipid-lowering effects [91]. Antiatherosclerotic effects of berberine have been explored via multiple animal and clinical studies. Previous findings have shown that berberine disrupts the NF-κB-mediated signaling pathway, and inhibits LDL oxidative modification, and cholesterol accumulation in macrophages. Recent studies indicate that berberine inhibits MMP-9 production by downregulating the expression of the P2X7 receptor [92]. This ultimately reduces extracellular matrix degradation and stabilizes atherosclerotic plaques. Several specific small-molecule antagonists including A740003, A804598, AZ10606120, GW791343, and JNJ47965567 form a structure that competes with the ATP binding site in the two adjacent subunits of the P2X7 receptor, hence inhibiting its activation [93].

#### 3.1.3 Other Drugs Targeting P2X7 Receptors

Colchicine effectively hinders pore formation induced by P2X7 receptors, thereby abrogating potassium ion flow from the cytoplasm [94, 95]. Colchicine exhibits broad anti-inflammatory effects by inhibiting microtubule formation, mitosis, leukocyte motility and the release of inflammatory cytokines. Colchicine inhibits the assembly of the *NLRP3*, thereby reducing the production of downstream IL-1β, IL-6, etc. [96]. Colchicine has been used gout, rheumatoid arthritis. In recent years, it has been increasingly used in the field of cardiovascular diseases [97]. Clinical trials of colchicine have been carried out in cardiovascular diseases such as acute pericarditis, atherosclerosis and acute myocardial infarction. In a randomized double-blind trial conducted in patients 30 days after myocardial infarction, it was demonstrated that 0.5 mg colchicine daily significantly reduced the risk of ischemic cardiovascular events due to atherosclerotic complications [98]. The COLCOT trial also showed that adjunctive use of low-dose colchicine after myocardial infarction can prevent inflammation [99]. A meta-analysis indicates that low-dose colchicine reduced the risk of major adverse cardiovascular events as well as that of myocardial infarction, stroke, and the need for coronary revascularization in a broad spectrum of patients with coronary disease [100].

Additionally, the P2X7 receptor is blocked by novel biological agents, including antibodies and nanoantibodies, with high specificity in various inflammatory models [101]. Pannexin-1 is associated with P2X7 receptor activation, hence, its inhibitors also block *NLRP3* inflammasome activation. Probenecid is a prevalent pannexin-1 antagonist. *In vitro* treatment of macrophages with probenecid reduces *NLRP3* inflammasome-dependent *IL-1β* secretion and inhibits P2X7 receptor signaling. Moreover, probenecid directly blocks the P2X7 receptor [102]. Pannexin-1 channel is a heptamer comprising a narrow extracellular domain (ECD), a tapered transmembrane domain (TMD), and an intracellular domain (ICD). Carbenoxolone (CBX) binds to residue 74 (W74) of the extracellular domain of the pannexin-1 channel and prevents ATP release [53]. Other inhibitors including glybenclamide, DIDS, and probenecid, block the pannexin-1 channel via this mechanism [103]. A previous study reported that CBX inhibits NF-κB pathway activation and downregulates *NLRP3* inflammasome expression and inflammatory cytokines [104].

#### 3.1.4 Drugs Targeting Kv1.3 Voltage-Gated Potassium Channels

Kv1.3 voltage-gated potassium channel is a primary potassium channel in macrophages, and its activation causes potassium ion outflow. *NLRP3* inflammasome is a downstream molecule of the Kv1.3 voltage-gated potassium channel. Kv1.3 is involved in apoptosis, migration, proliferation, and activation of macrophages. Blocking Kv1.3 with Margatoxin, its specific inhibitor prevents macrophages from converting into foam cells in atherosclerosis [105]. Previous findings indicate that *NLRP3*, ASC, and caspase-1 expression are significantly upregulated in colitis; inhibition of the NLRP3 inflammasome pathway effectively reduces the severity of colitis [106]. Recent studies reveal that PAP-1 (a Kv1.3 channel-specific blocker) downregulates Kv1.3 expression in macrophages in mice with colitis and inhibits macrophage activation [107]. Moreover, PAP-1 effectively inhibits the expression of *NLRP3*, *ASC*, caspase-1p20 and *IL-1β*. Studies on cerebral ischemia-reperfusion injury have documented that Kv1.3 blockers suppress cleavage of caspase-1 and *IL-1β*, thereby blocking *IL-1β* release and inhibiting the positive feedback signal of *NF-κB* on *NLRP3* inflammasome activation [108].

#### 3.1.5 K2P Channel Modulators

K2P channel modulators have been extensively investigated in recent years. Xiao-yan Wu *et al*. [109] used various K2P channel modulators, including quinine, fluoxetine, DCPIB, ML365, ML335, and TKDC to evaluate their effects on K2P channels. As a result, quinine and fluoxetine were non-selective and weak blockers of all K2P channels. ML365 showed a high selective inhibitory effect on TWIK2 via dose-dependent inhibition of ATP-induced *NLRP3* inflammasome activation. Moreover, ML365 administration decreased the levels of *IL-1β* and active caspase-1p20 subunit, as well as ameliorated LPS-induced endotoxin shock. Three mutations were introduced into the C-terminus of TWIK2 to upregulate TWIK2 expression in the cell membrane, which may influence its effect.

### 3.2 Other Related Drug Targets

At present, clinical treatment of *NLRP3*-related diseases primarily targets *IL-1β*. For instance, *IL-1β* antibodies and recombinant *IL-1β* antagonists include canakinumab and anakinra. Nonetheless, *IL-1β* is ubiquitously secreted, therefore, these inhibitors are relatively nonspecific, with low efficacy, and potentially result in immunosuppression [110].

β-hydroxybutyrate (BHB) is a ketone metabolite that inhibits *NLRP3* inflammasome activation [111, 112]. A previous study revealed that incubation of BHB with the *NLRP3* activator ATP dose-dependently abrogated a decrease in intracellular potassium concentration [113]. Moreover, BHB reduces oligomerization of ASCs and effectively inhibits *IL-1β* and *IL-18* production in human monocytes. These findings show that pharmacological or dietary agents that increase BHB levels can relieve the severity of *NLRP3*-mediated chronic inflammatory disease. This is a potential mechanism for inflammation inhibition through a ketogenic diet.

Studies have shown that several small molecular compounds including MCC950, BHB, and Bay 11-7082 inhibit NLRP3 inflammasome activation *in vitro*. Nonetheless, the majority of these inhibitors are relatively non-specific with low efficacy. **Supplementary Table 2** presents recent pharmacological targets and associated clinical trials of *NLRP3* inflammasome pathway inhibitors.

## 4. Perspective

Incidence of atherosclerosis is projected to increase owing to increase in aging population and current changes in lifestyle. Studies report that atherosclerosis is a chronic inflammatory disease, associated with a variety of adverse cardiovascular events. NLRP3 inflammasome is an important component of innate immune system that links lipid metabolism to inflammation. Lipid deposition, ox-LDL, macrophage transformation into foam cells and other events associated with atherosclerosis interact with the NLRP3 inflammasome to promote progression of the disease. Initiation and activation of the NLRP3 inflammasome is a two-signal model in which potassium outflow is an indispensable process in signal 2. Only few drugs have been developed that target NLRP3 inflammasome due to the unknown mechanism of potassium outflow and activation of the NLRP3 inflammasome.

Currently, most drugs against NLRP3 inflammasome target IL-1β and NLRP3 proteins. These have high efficacy against inflammation and few side effects. Canakinumab and MCC950 are widely studied drugs targeting NLRP3 inflammasome. MCC950 has high therapeutic potential due to the low concentration required to inhibit NLRP3 and has low toxicity. Studies report that P2X7 receptors, pannexin-1, K2P channels, and GSDMD are associated with NLRP3 inflammasome. Nek7 and KATP channels and potassium voltage-gated channels such as KCNA3 and KCNB2 are implicated in activation of NLRP3 inflammasome. Drugs that inhibit potassium outflow events at these targets include the sulfonylurea compound glibenclamide, P2X7 receptor antagonists such as berberine and colchicine, antibodies, pannexin-1 antagonist, Kv1.3 voltage-gated potassium channel antagonists including Margatoxin and PAP-1, as well as K2P channel modulators such as quinine and fluoxetine. Although the effects of most drugs have been explored *in vitro* or in animal studies, it is important to note that only glibenclamide’s effect on NLRP3 inflammasome activation has been extensively studied, and the inhibition of these drugs in humans has not been verified in clinical trials also. In conclusion, it is imperative to explore the specific mechanism of NLRP3 inflammasome and atherosclerosis. Further research should also explore the potential therapeutic effects of compounds targeting NLRP3 inflammasome.

## Supplementary Material

Supplementary material associated with this article can be found, in the online version, at https://doi.org/10.31083/j.rcm2308268.
