# Supplementary material for: NLRP3 Inflammasome as a Therapeutic Target for Atherosclerosis: A Focus on Potassium Outflow
Source: Rev Cardiovasc Med. 2022 Jul 22;23(8):268. doi: 10.31083/j.rcm2308268 (PMC11266955; doi:10.31083/j.rcm2308268)
Supplement: Supplementary file 1 [file 2153-8174-23-8-268-s1.docx]

**Table 1.** Summary of other NLRP3 inflammasome activation events

| Other NLRP3 inflammasome activation events | Main mechanism/pathway/evidence |
| --- | --- |
| Calcium mobilization | The mechanism by which calcium ion mobilization induces NLRP3 inflammasome activation is also unclear and its role remains controversial. BAPTA, a calcium chelator, inhibits NLRP3 inflammasome activation and IL-1β processing[1]. In addition, a study[2]showed that 2-APB is a widely used IP3R inhibitor that inhibits the activation of inflammasome independent of its expected effect on calcium homeostasis. Therefore, the role of calcium mobilization in NLRP3 inflammasome activation needs to be further determined. The potassium ion outflow can be regulated by counterions flowing into the plasma membrane as calcium ion. Thus, calcium flux and potassium outflow are often found to coordinate in NLRP3 activation. Increased intracellular calcium ion concentration can be achieved in a variety of ways[3]:(1) ATP induces the opening of cell membrane P2X7 receptor; (2) Calcium channel such as TRPM2, TRPM7 and TRPV2 are induced to open by crystal, charged liposome and cell swelling. (3) Pore-forming toxins and membrane attack complexes directly induce calcium ion to cross the damaged membrane; (4) Increased intracellular calcium ion promotes the release of more calcium ion from endoplasmic reticulum(ER) through IP3 pathway; (5) G-protein-coupled receptors (GPCR), such as calcium sensitive receptors (CaSR) and GPRC6A, can also trigger ER through PLC activation, IP3 generation and IP3R opening to induce calcium ion release. Too much calcium leads to mitochondrial calcium overload and mitochondrial damage, which ultimately leads to the production of mtROS and activation of NLRP3 inflammasome. |
| Chloride outflow | The increase of extracellular chloride ion concentration inhibits IL-1β release; Chloride channel inhibitors reduce IL-1βrelease; CLIC1 and CLIC4 are both responsive to LPS stimulation, and NLRP3 agonists such as ROS can promote CLIC plasma membrane translocation, thereby promoting chloride outflow and NLRP3 activation in macrophages, and the reduction of these two channels reduces ASC formation in general [4]. WNK1 balances intracellular ions (including sodium ion, potassium ion and chloride ion) by activating positive ion -chloride ion-cotransporter, thus inhibiting the activation of NLRP3 inflammasome. A experiment[5]found that NLRP3 inflammasome was strongly activated only when intracellular chloride and potassium ions decreased simultaneously. CLICs-dependent chloride outflow regulates Nek7-NLRP3 complex formation to promote NLRP3 inflammasome activation[6]. |
| Sodium inflow | The mechanism that sodium influx activates NLRP3 inflammasome is related to potassium outflow. Epithelial sodium channel (ENaC) -mediated sodium inflow makes cells more susceptible to potassium efflux in response to ATP stimulation, so the possible mechanism is dependent on potassium efflux to activate NLRP3 inflammasome as modulators. Inhibition of ENaC was also observed to inhibit the formation of ASC spots, decrease caspase-1 activity, and decrease cytokine secretion[7]. Another possible mechanism is the mitochondrial Na/Ca exchanger (NCLX), which maintains intracellular calcium concentration and mitochondrial intimal homeostasis through ion exchange, preventing mitochondrial damage, ROS production and other events that activate NLRP3 inflammasome[8]. In conclusion, sodium influx alone is insufficient to induce NLRP3 inflammasome activation. |
| Mitochondrial oxidative damage | Almost all NLRP3 activation signals cause mitochondrial dysfunction, resulting in ROS production and mtDNA release. Mitochondrial transcription factor A (TFAM) binds to mtDNA to promote its stability, and TFAM defect shows inactive caspase-1 and IL-1β. Moreover, the presence of hydrogen peroxide does not restore the NLRP3 inflammasome activation in TFAM deficient cells, suggesting that mtROS triggers NLRP3 inflammasome activation by promoting ox-mtDNA production. Meanwhile, endogenous ox-mtDNA co-located with ASC-containing inflammasome complex after NLRP3 activator treatment[9]. A experiment[10]has shown that ROS was a potential NLRP3 inflammasome induction signal and could directly regulate GSDMD cleavage. |
| Endoplasmic Reticulum stress(ERS) [11; 12] | ERS directly promotes NF-κB pathway. Activated NLRP3 locates in the mitochondria-associated endoplasmic reticulum (MAM), and the close proximity between ER and mitochondria in the MAM promotes NLRP3 interaction between the two. ERS sensor IRE1 is a key regulator of NLRP3 inflammasome activation. Activated IRE1α promotes mitochondrial ROS release, redistributes NLRP3 from ER to mitochondria, and triggers caspase-2 cleavage and recruitment to mitochondria. ERS sensor PERK delivers ROS-mediated ERS-dependent apoptosis as signal 2 to activate NLRP3 inflammasome. After ER activation, calcium ion flows into mitochondria, resulting in calcium ion overload and instability of mitochondria, thus activating NLRP3 through mitochondrial injury mechanism. Cholesterol accumulation in the ER, a major site of lipid synthesis, also activates NLRP3 inflammasome. Overall, ERS can lead to activation of NLRP3 inflammasome, and ERS can also be induced by NLRP3 inflammasome. Mitochondrial ROS bridge the interaction between ERS and NLRP3 inflammasome. |
| Dispersed Trans-Golgi Network(TGN) | Five NLRP3 agonists with different structures: Nigeritin, ATP, bacitracin, imiquimote, and CL097 can trigger the decomposition of TGN, on which NLRP3 forms different spots[13]. In a vitro experiment[14]observed that NLRP3 could be activated after treatment with Nigeritin, and it was found that NLRP3 first formed multiple small spots on the dispersed TGN (dTGN) before binding to a large spot with ASC adaptor protein. In contrast, the cis-medial Golgi remained intact and did not co-locate with NLRP3. In response to NLRP3 activator, dTGN formed before NLRP3 spots, suggesting that it promoted recruitment and aggregation of NLRP3. Researchers identified phosphatidylinositol 4-phosphate (PtdIns4P) as a negatively charged phospholipid that mediated NLRP3 recruitment to dTGN. Potassium efflux was also found to be necessary for NLRP3 to recruit dTGN after being stimulated by Nigeritin. The researchers suggest that potassium outflow may promote ion binding between NLRP3 and PtdIns4P. In contrast, imiquimod and CL097 did not rely on potassium outflow to induce TGN recruitment and activation of NLRP3. |
| Lysosomal membrane rupture | NLRP3 activation signals may cause lysosome instability and rupture. Studies[15]have shown that lysosomal rupture was a weak inducer of caspase-1 and could activate NLRP3 inflammasome by triggering cathepsin dependent protein degradation. Cathepsin B released by lysosome breakdown[16]binds directly to NLRP3 inflammasome and promotes its activation. Lysosome damage can also activate potassium outflow and calcium mobilization[17]. The Ca^2＋^-CaMKII-TAK1-JNK pathway activated by lysosome breakdown regulates NLRP3 inflammasome activation by promoting ASC oligomerization[18]. |
| Cell autophagy | Autophagy interacts with NLRP3 inflammasome[19]. Autophagy can negatively regulate NLRP3 inflammasome activation by removing endogenous inflammasome activation signals (e.g., damaged mitochondria), removing inflammasome components (NLRP3, ASC, etc.) and cytokines, which can prevent excessive inflammatory responses. NLRP3 can reduce autophagy by NACHT interaction with proteins involved in autophagy. Activation of caspase-1 may lead to inhibition of autophagy induction, thereby increasing the inflammatory response. In response to NLRP3 activator, increased IL-1β secretion was observed in autophagy-deficient macrophages accompanied by accumulation of damaged mitochondria. Meanwhile, NF-κB selectively induces SQSTM1 expression after NLRP3 and pro-IL-1β expression in LPS-treated macrophages. SQSTM1 ablation leads to impaired autophagy, which further increases IL-1β secretion through NLRP3 inflammasome activation, suggesting that SQSTM1-dependent autophagy plays a key role in inhibiting NF-κB activated IL-1β secretion[20]. |
| Metabolic disorders[21; 22] | There have been a number of findings suggesting close interactions between NLRP3 and metabolism. Metabolites have been shown to activate NLPR3, and impaired mitochondria have been shown to be involved in NLRP3 function also. Chronic hyperglycemia leads to the release of an NLRP3 agonist; ROS and uric acid levels in obese individuals can be used as NLRP3 activators. IL-1β produced after activation of NLRP3 inflammasome in turn affects many metabolic signals: IL-1β damages islet cells, reduces the expression and activation of downstream signaling components of insulin receptors, and leads to decreased insulin-induced glucose transport and fat production, ultimately leading to decreased lipid content in adipocytes. IL-1β also interferes with adipocyte differentiation. |

**Table 2.** Recent pharmacological targets and associated clinical trials of NLRP3 inflammasome pathway inhibitors

| Common drugs | Target/Mechanism | Clinical trials |
| --- | --- | --- |
| Canakinumab | Therapeutic monoclonal antibody targeting IL-1β | Several CANTOS (Canakinumab anti-inflammatory Outcomes Study)[23; 24; 25]have shown that Canakinumab reduces adverse cardiovascular outcomes in atherosclerosis. |
| MCC950 | MCC950 highly specific non-covalent interaction with or near the WalkerB motif in the NACHT domain of NLRP3 inhibits its ATPase activity and keeps its closed and inactive conformation, and blocks ASC oligomerization[26; 27]. MCC950 destroys the binding of Nek7 to NLRP3[28] and targets chloride outflow. | No large clinical trial has been found. Most of the experiments were conducted on mice, and MCC950 have been proved to be effective in a variety of cardiovascular inflammatory disease models, such as myocardial fibrosis, myocardial infarction[29], aortic aneurysm, aortic dissection[30], etc. |
| CY-09 | CY-09 specifically binds to the WalkerA motif in the NACHT domain of NLRP3 to inhibit ATPase activity and ASC oligomerization, and it inhibits LPS-induced non-classical NLRP3 activation[31], which is reversible. | No large clinical trial has been found. Most of the experiments were in mice and rarely demonstrated beneficial effects in cardiovascular inflammatory disease. |
| OLT1177/Dapansutrile | OLT1177 specifically interacts with NLRP3 protein to inhibit ATPase activity and prevent NLRP3 oligomerization. | A phase 1 of clinical trial about OLT1177 orally administered to healthy subjects proved that it was safe and well tolerated, without observing any toxicity to clinical, hematological or organs at any test dose, which achieved meaningful exposure and a long half-life[32]. A phase 1B trial[33]in patients with systolic heart failure also showed good safety and tolerability. A phase 2A trial[34]in patients with gout showed relief of joint pain and swelling with few adverse events. |
| Tranilast | Tranilast significantly promotes NLRP3 ubiquitination of K63 connection mainly in macrophages. By inhibiting the binding of Nek7 to NLRP3, the assembly of NLRP3 inflammasome is restricted. It also inhibits ASC oligomerization and NLRP3 inflammasome activation[35]. | No large clinical trial has been found. Experiments on mice showed that Tranilast could inhibit the occurrence and development of atherosclerosis and limit the formation of necrotic core in plaques, and it also improves myocardial infarction[36]. |
| Oridonin | Oridonin directly targets NLRP3 by covalently binding to the NACHT domain. The endogenous Nek7-NLRP3 and NLRP3-ASC interactions are also blocked. Its effect is irreversible[37]. | No large clinical trial has been found. Experiments[38]on mice showed that it can alleviate myocardial fibrosis and cardiac remodeling induced by myocardial infarction and improve a variety of inflammatory models. |
| Colchicine | Colchicine inhibits the expression of pyrin gene and also inhibits tubulin, interfering with the cytoplasmic co-localization of inflammasome and thus inhibiting the assembly of NLRP3 inflammasome. Colchicine directly inhibits pore formation induced by caspase-1 and P2X7 receptors. It also inhibits foam cell formation induced by ox-LDL and cholesterol crystals and blocks NLRP3 inflammasome initiation[39]. | Jean-Claude Tardif et al. demonstrated that 0.5mg colchicine per day significantly reduced the risk of ischemic cardiovascular events from atherosclerotic complications in a randomized double-blind trial of approximately 4500 patients within 30 days after myocardial infarction[40]. Similar subsequent trial[41]has also shown that adjuvant use of low-dose colchicine early after myocardial infarction can prevent inflammation. |
| Parthenolide | The NF-κB pathway is directly inhibited by parthenolide. It inactivates caspase-1 by alkylation of cysteine residues at position 285 of the p20 subunit and inhibits NLRP3 ATPase activity in a dose-dependent manner, as well as NLRP3 inflammasome induced apoptosis[42]. | No large clinical trial has been found. Clinical trials are mostly cancer-related. |
| Bay 11-7082 | Bay 11-7082 selectively inhibits the NLRP3 inflammasome activation pathway. It inhibits the NF-κB pathway by blocking the kinase activity of IKKβ and inhibits the ATPase activity of NLRP3 in a dose-dependent manner and inhibits the NLRP3 inflammasome by alkylation of the target by Michael addition at its own C-2[42]. | No large clinical trial has been found. Clinical trials are mostly related to pregnancy and childbirth. |
| 3,4-Methylenedioxy-β-nitrostyrene (MNS) | MNS specifically binds to the NOD and LRR domains of NLRP3, and its nitrostyrene side chain binds to NLRP3 cysteine residues, directly inhibiting NLRP3 ATPase activity and thus NLRP3 mediated ASC spot formation[43]. | No large clinical trial has been found. |

**References**

[1] M.A. Katsnelson, L.G. Rucker, H.M. Russo, and G.R. Dubyak, K+ efflux agonists induce NLRP3 inflammasome activation independently of Ca2+ signaling. J Immunol 194 (2015) 3937-52.

[2] A.G. Baldwin, J. Rivers-Auty, M.J.D. Daniels, C.S. White, C.H. Schwalbe, T. Schilling, H. Hammadi, P. Jaiyong, N.G. Spencer, H. England, N.M. Luheshi, M. Kadirvel, C.B. Lawrence, N.J. Rothwell, M.K. Harte, R.A. Bryce, S.M. Allan, C. Eder, S. Freeman, and D. Brough, Boron-Based Inhibitors of the NLRP3 Inflammasome. Cell Chem Biol 24 (2017) 1321-1335 e5.

[3] T. Gong, Y. Yang, T. Jin, W. Jiang, and R. Zhou, Orchestration of NLRP3 Inflammasome Activation by Ion Fluxes. Trends Immunol 39 (2018) 393-406.

[4] R. Domingo-Fernandez, R.C. Coll, J. Kearney, S. Breit, and L.A.J. O'Neill, The intracellular chloride channel proteins CLIC1 and CLIC4 induce IL-1beta transcription and activate the NLRP3 inflammasome. J Biol Chem 292 (2017) 12077-12087.

[5] L. Mayes-Hopfinger, A. Enache, J. Xie, C.L. Huang, R. Kochl, V.L.J. Tybulewicz, T. Fernandes-Alnemri, and E.S. Alnemri, Chloride sensing by WNK1 regulates NLRP3 inflammasome activation and pyroptosis. Nat Commun 12 (2021) 4546.

[6] T.T. Tang, X.T. Lang, C.F. Xu, X.Q. Wang, T. Gong, Y.Q. Yang, J. Cui, L. Bai, J. Wang, W. Jiang, and R.B. Zhou, CLICs-dependent chloride efflux is an essential and proximal upstream event for NLRP3 inflammasome activation. Nature Communications 8 (2017).

[7] T. Scambler, H.H. Jarosz-Griffiths, S. Lara-Reyna, S. Pathak, C. Wong, J. Holbrook, F. Martinon, S. Savic, D. Peckham, and M.F. McDermott, ENaC-mediated sodium influx exacerbates NLRP3-dependent inflammation in cystic fibrosis. Elife 8 (2019).

[8] Y. Zu, L.J. Wan, S.Y. Cui, Y.P. Gong, and C.L. Li, The mitochondrial Na+/Ca2+ exchanger may reduce high glucose-induced oxidative stress and nucleotide-binding oligomerization domain receptor 3 inflammasome activation in endothelial cells. J Geriatr Cardiol 12 (2015) 270-278.

[9] Z. Zhong, S. Liang, E. Sanchez-Lopez, F. He, S. Shalapour, X.J. Lin, J. Wong, S. Ding, E. Seki, B. Schnabl, A.L. Hevener, H.B. Greenberg, T. Kisseleva, and M. Karin, New mitochondrial DNA synthesis enables NLRP3 inflammasome activation. Nature 560 (2018) 198-203.

[10] Y. Wang, P. Shi, Q. Chen, Z. Huang, D. Zou, J. Zhang, X. Gao, and Z. Lin, Mitochondrial ROS promote macrophage pyroptosis by inducing GSDMD oxidation. J Mol Cell Biol 11 (2019) 1069-1082.

[11] Y. Zhou, Z. Tong, S. Jiang, W. Zheng, J. Zhao, and X. Zhou, The Roles of Endoplasmic Reticulum in NLRP3 Inflammasome Activation. Cells 9 (2020).

[12] T. Ji, Y. Han, W. Yang, B. Xu, M. Sun, S. Jiang, Y. Yu, Z. Jin, Z. Ma, Y. Yang, and W. Hu, Endoplasmic reticulum stress and NLRP3 inflammasome: Crosstalk in cardiovascular and metabolic disorders. J Cell Physiol (2019).

[13] K.W. Chen, D. Boucher, and P. Broz, Divide to conquer: NLRP3 is activated on dispersed trans-Golgi network. Cell Res 29 (2019) 181-182.

[14] J. Chen, and Z.J. Chen, PtdIns4P on dispersed trans-Golgi network mediates NLRP3 inflammasome activation. Nature 564 (2018) 71-76.

[15] H. Lima, Jr., L.S. Jacobson, M.F. Goldberg, K. Chandran, F. Diaz-Griffero, M.P. Lisanti, and J. Brojatsch, Role of lysosome rupture in controlling Nlrp3 signaling and necrotic cell death. Cell Cycle 12 (2013) 1868-78.

[16] M. Bruchard, G. Mignot, V. Derangere, F. Chalmin, A. Chevriaux, F. Vegran, W. Boireau, B. Simon, B. Ryffel, J.L. Connat, J. Kanellopoulos, F. Martin, C. Rebe, L. Apetoh, and F. Ghiringhelli, Chemotherapy-triggered cathepsin B release in myeloid-derived suppressor cells activates the Nlrp3 inflammasome and promotes tumor growth. Nat Med 19 (2013) 57-64.

[17] J.D. Schilling, Dousing fire with gasoline: interplay between lysosome damage and the NLRP3 inflammasome. Focus on "NLRP3 inflammasome signaling is activated by low-level lysosome disruption but inhibited by extensive lysosome disruption: roles for K+ efflux and Ca2+ influx". Am J Physiol-Cell Ph 311 (2016) C81-C82.

[18] M. Okada, A. Matsuzawa, A. Yoshimura, and H. Ichijo, The lysosome rupture-activated TAK1-JNK pathway regulates NLRP3 inflammasome activation. J Biol Chem 289 (2014) 32926-36.

[19] M. Biasizzo, and N. Kopitar-Jerala, Interplay Between NLRP3 Inflammasome and Autophagy. Frontiers in Immunology 11 (2020).

[20] Y. Xu, J. Shen, and Z. Ran, Emerging views of mitophagy in immunity and autoimmune diseases. Autophagy 16 (2020) 3-17.

[21] M. Haneklaus, and L.A.J. O'Neill, NLRP3 at the interface of metabolism and inflammation. Immunological Reviews 265 (2015) 53-62.

[22] D. Jiang, S. Chen, R. Sun, X. Zhang, and D. Wang, The NLRP3 inflammasome: Role in metabolic disorders and regulation by metabolic pathways. Cancer Lett 419 (2018) 8-19.

[23] P.M. Ridker, B.M. Everett, T. Thuren, J.G. MacFadyen, W.H. Chang, C. Ballantyne, F. Fonseca, J. Nicolau, W. Koenig, S.D. Anker, J.J.P. Kastelein, J.H. Cornel, P. Pais, D. Pella, J. Genest, R. Cifkova, A. Lorenzatti, T. Forster, Z. Kobalava, L. Vida-Simiti, M. Flather, H. Shimokawa, H. Ogawa, M. Dellborg, P.R.F. Rossi, R.P.T. Troquay, P. Libby, R.J. Glynn, and C.T. Group, Antiinflammatory Therapy with Canakinumab for Atherosclerotic Disease. N Engl J Med 377 (2017) 1119-1131.

[24] B.M. Everett, J.G. MacFadyen, T. Thuren, P. Libby, R.J. Glynn, and P.M. Ridker, Inhibition of Interleukin-1beta and Reduction in Atherothrombotic Cardiovascular Events in the CANTOS Trial. J Am Coll Cardiol 76 (2020) 1660-1670.

[25] P.M. Ridker, J.G. MacFadyen, T. Thuren, P. Libby, and C.T. Grp, Residual inflammatory risk associated with interleukin-18 and interleukin-6 after successful interleukin-1 beta inhibition with canakinumab: further rationale for the development of targeted anti-cytokine therapies for the treatment of atherothrombosis. Eur Heart J 41 (2020) 2153-2163.

[26] R.C. Coll, J.R. Hill, C.J. Day, A. Zamoshnikova, D. Boucher, N.L. Massey, J.L. Chitty, J.A. Fraser, M.P. Jennings, A.A.B. Robertson, and K. Schroder, MCC950 directly targets the NLRP3 ATP-hydrolysis motif for inflammasome inhibition. Nat Chem Biol 15 (2019) 556-559.

[27] A. Tapia-Abellan, D. Angosto-Bazarra, H. Martinez-Banaclocha, C. de Torre-Minguela, J.P. Ceron-Carrasco, H. Perez-Sanchez, J.I. Arostegui, and P. Pelegrin, MCC950 closes the active conformation of NLRP3 to an inactive state. Nat Chem Biol 15 (2019) 560-564.

[28] Y. Zhang, X. Lv, Z. Hu, X. Ye, X. Zheng, Y. Ding, P. Xie, and Q. Liu, Protection of Mcc950 against high-glucose-induced human retinal endothelial cell dysfunction. Cell Death Dis 8 (2017) e2941.

[29] R. Gao, H. Shi, S. Chang, Y. Gao, X. Li, C. Lv, H. Yang, H. Xiang, J. Yang, L. Xu, and Y. Tang, The selective NLRP3-inflammasome inhibitor MCC950 reduces myocardial fibrosis and improves cardiac remodeling in a mouse model of myocardial infarction. Int Immunopharmacol 74 (2019) 105575.

[30] P. Ren, D. Wu, R. Appel, L. Zhang, C. Zhang, W. Luo, A.A.B. Robertson, M.A. Cooper, J.S. Coselli, D.M. Milewicz, Y.H. Shen, and S.A. LeMaire, Targeting the NLRP3 Inflammasome With Inhibitor MCC950 Prevents Aortic Aneurysms and Dissections in Mice. J Am Heart Assoc 9 (2020) e014044.

[31] H. Jiang, H. He, Y. Chen, W. Huang, J. Cheng, J. Ye, A. Wang, J. Tao, C. Wang, Q. Liu, T. Jin, W. Jiang, X. Deng, and R. Zhou, Identification of a selective and direct NLRP3 inhibitor to treat inflammatory disorders. J Exp Med 214 (2017) 3219-3238.

[32] C. Marchetti, B. Swartzwelter, F. Gamboni, C.P. Neff, K. Richter, T. Azam, S. Carta, I. Tengesdal, T. Nemkov, A. D'Alessandro, C. Henry, G.S. Jones, S.A. Goodrich, J.P. St Laurent, T.M. Jones, C.L. Scribner, R.B. Barrow, R.D. Altman, D.B. Skouras, M. Gattorno, V. Grau, S. Janciauskiene, A. Rubartelli, L.A.B. Joosten, and C.A. Dinarello, OLT1177, a beta-sulfonyl nitrile compound, safe in humans, inhibits the NLRP3 inflammasome and reverses the metabolic cost of inflammation. Proc Natl Acad Sci U S A 115 (2018) E1530-E1539.

[33] G.F. Wohlford, B.W. Van Tassell, H.E. Billingsley, D. Kadariya, J.M. Canada, S. Carbone, V.L. Mihalick, A. Bonaventura, A. Vecchie, J.G. Chiabrando, E. Bressi, G. Thomas, A.C. Ho, A.A. Marawan, M. Dell, C.R. Trankle, J. Turlington, R. Markley, and A. Abbate, Phase 1B, Randomized, Double-Blinded, Dose Escalation, Single-Center, Repeat Dose Safety and Pharmacodynamics Study of the Oral NLRP3 Inhibitor Dapansutrile in Subjects With NYHA II-III Systolic Heart Failure. J Cardiovasc Pharmacol 77 (2020) 49-60.

[34] V. Kluck, T. Jansen, M. Janssen, A. Comarniceanu, M. Efde, I.W. Tengesdal, K. Schraa, M.C.P. Cleophas, C.L. Scribner, D.B. Skouras, C. Marchetti, C.A. Dinarello, and L.A.B. Joosten, Dapansutrile, an oral selective NLRP3 inflammasome inhibitor, for treatment of gout flares: an open-label, dose-adaptive, proof-of-concept, phase 2a trial. Lancet Rheumatol 2 (2020) e270-e280.

[35] S. Chen, Y. Wang, Y. Pan, Y. Liu, S. Zheng, K. Ding, K. Mu, Y. Yuan, Z. Li, H. Song, Y. Jin, and J. Fu, Novel Role for Tranilast in Regulating NLRP3 Ubiquitination, Vascular Inflammation, and Atherosclerosis. J Am Heart Assoc 9 (2020) e015513.

[36] D. Qu, H. Guo, and Y. Xu, Effects of Tranilast on Inflammasome and Macrophage Phenotype in a Mouse Model of Myocardial Infarction. J Interferon Cytokine Res 41 (2021) 102-110.

[37] H.B. He, H. Jiang, Y. Chen, J. Ye, A.L. Wang, C. Wang, Q.S. Liu, G.L. Liang, X.M. Deng, W. Jiang, and R.B. Zhou, Oridonin is a covalent NLRP3 inhibitor with strong anti-inflammasome activity. Nature Communications 9 (2018).

[38] R.F. Gao, X. Li, H.Y. Xiang, H. Yang, C.Y. Lv, X.L. Sun, H.Z. Chen, Y. Gao, J.S. Yang, W. Luo, Y.Q. Yang, and Y.H. Tang, The covalent NLRP3-inflammasome inhibitor Oridonin relieves myocardial infarction induced myocardial fibrosis and cardiac remodeling in mice. Int Immunopharmacol 90 (2021) 107133.

[39] G.J. Martinez, D.S. Celermajer, and S. Patel, The NLRP3 inflammasome and the emerging role of colchicine to inhibit atherosclerosis-associated inflammation. Atherosclerosis 269 (2018) 262-271.

[40] J.C. Tardif, S. Kouz, D.D. Waters, O.F. Bertrand, R. Diaz, A.P. Maggioni, F.J. Pinto, R. Ibrahim, H. Gamra, G.S. Kiwan, C. Berry, J. Lopez-Sendon, P. Ostadal, W. Koenig, D. Angoulvant, J.C. Gregoire, M.A. Lavoie, M.P. Dube, D. Rhainds, M. Provencher, L. Blondeau, A. Orfanos, P.L. L'Allier, M.C. Guertin, and F. Roubille, Efficacy and Safety of Low-Dose Colchicine after Myocardial Infarction. N Engl J Med 381 (2019) 2497-2505.

[41] N. Bouabdallaoui, J.C. Tardif, D.D. Waters, F.J. Pinto, A.P. Maggioni, R. Diaz, C. Berry, W. Koenig, J. Lopez-Sendon, H. Gamra, G.S. Kiwan, L. Blondeau, A. Orfanos, R. Ibrahim, J.C. Gregoire, M.P. Dube, M. Samuel, O. Morel, P. Lim, O.F. Bertrand, S. Kouz, M.C. Guertin, P.L. L'Allier, and F. Roubille, Time-to-treatment initiation of colchicine and cardiovascular outcomes after myocardial infarction in the Colchicine Cardiovascular Outcomes Trial (COLCOT). Eur Heart J 41 (2020) 4092-4099.

[42] C. Juliana, T. Fernandes-Alnemri, J. Wu, P. Datta, L. Solorzano, J.W. Yu, R. Meng, A.A. Quong, E. Latz, C.P. Scott, and E.S. Alnemri, Anti-inflammatory compounds parthenolide and Bay 11-7082 are direct inhibitors of the inflammasome. J Biol Chem 285 (2010) 9792-9802.

[43] Y. He, S. Varadarajan, R. Munoz-Planillo, A. Burberry, Y. Nakamura, and G. Nunez, 3,4-methylenedioxy-beta-nitrostyrene inhibits NLRP3 inflammasome activation by blocking assembly of the inflammasome. J Biol Chem 289 (2014) 1142-50.
